# Pulmonary infection due to *Balamuthia mandrillaris* in the southwestern United States: not all miliary disease is tuberculosis and coccidioidomycosis

**DOI:** 10.1128/asmcr.00078-24

**Published:** 2025-05-09

**Authors:** N. B. Price, H. Pariury, J. Papic, M. R. Anthony, W. Lainhart, K. W. Shehab

**Affiliations:** 1 Department of Pediatrics, Division of Pediatric Infectious Diseases, University of Arizona College of Medicine, Tucson, Arizona, USA; 2 Department of Pediatrics, Division of Pediatric Hematology Oncology, University of Arizona College of Medicine, Tucson, Arizona, USA; 3 Department of Surgery, University of Arizona College of Medicine, Tucson, Arizona, USA; 4 Department of Pathology and Laboratory Medicine, University of Arizona College of Medicine, Tucson, Arizona, USA; Pattern Bioscience, Austin, Texas, USA

**Keywords:** amoeba, balamuthiasis

## Abstract

**Background:**

*Balamuthia mandrillaris* is a rare cause of skin and/or central nervous system (CNS) infection with a high mortality rate. Dissemination to other organs, including the lungs, is rare. Despite worldwide distribution, this disease entity is underrecognized. Delay in diagnosis may contribute to high mortality.

**Case Summary:**

A 17-year-old Hispanic male living in Arizona on immunosuppressive therapy for Castleman’s disease presented with new cutaneous lesions and cough and was found to have miliary pulmonary disease on chest imaging. He was suspected of having disseminated coccidioidomycosis or tuberculosis, but skin biopsy revealed amebae. Skin and lung biopsy tissue confirmed the presence of *Balamuthia mandrillaris* by real-time PCR. Additional imaging revealed CNS involvement. Despite initial good appearance and initiation of multidrug antimicrobial therapy, he developed multiorgan failure and died within 17 days of presentation.

**Conclusion:**

This is the first published case of miliary lung disease caused by *Balamuthia mandrillaris*. Infection can affect multiple organ systems with poor outcomes even in the setting of early antimicrobial therapy. *Balamuthia* should be considered in patients with miliary lung disease.

## INTRODUCTION

*Balamuthia mandrillaris* is a free-living ameba that has been found in soil and water specimens throughout the world (1). Originally thought to be non-pathogenic, *B. mandrillaris* was implicated in the fatal infection of a mandrill at the San Diego Zoo in 1986. Once discovered, retrospective evaluation of some fatal encephalitis cases with unidentified amebae was confirmed to be due to *Balamuthia* (2).

The type and severity of *Balamuthia mandrillaris* disease vary and can affect both immunocompetent and immunocompromised hosts. In the literature, it appears that initial cutaneous disease with higher survivability tends to occur in China and South America, although cases can still progress to develop central nervous system (CNS) infection with a high fatality rate (3, 4). However, in the United States, published cases tend to suggest a more rapid disease progression and high fatality rate, even with antimicrobial therapy (5). The reason for these geographic differences in case presentation and survivability is unknown, but could possibly be attributed to differences in diagnosis and reporting. It is unclear how initial infection and invasion with *B. mandrillaris* occur. Other free-living ameba such as *Naegleria* invade via the olfactory nerve after freshwater exposure, and *Acanthamoeba* by hematogenous spread after soil or water exposure. Entry of *Balamuthia* via skin or respiratory tract with hematogenous spread, or by direct invasion via the olfactory nerve is postulated to occur (6–9). Pulmonary balamuthiasis is rare, but cases have been described (2, 8, 10–24). Many cases of balamuthiasis in the United States have occurred in the southwestern region (5). With both symptomatic and geographic overlap of this pathogen with *Mycobacterium tuberculosis* and *Coccidioides immitis/posadasii*, which are much more common (25, 26), as well as overlap with other medical conditions, delays in both diagnosis and treatment of balamuthiasis are common (3).

Herein, we describe the first patient to present with miliary pulmonary balamuthiasis and summarize the data of other *B. mandrillaris* cases with confirmed or probable pulmonary involvement.

## CASE PRESENTATION

A 17-year-old Hispanic male presented with skin lesions and miliary pulmonary disease on plain radiograph of the chest. The patient had a remote history of cutaneous mycosis fungoides and was recently treated with steroids and rituximab for HHV-8-negative multicentric Castleman’s disease, diagnosed approximately 6 months prior to presentation.

Seven years prior to presentation, the patient developed a bruise-like rash on his posterolateral left thigh after minor trauma. Over the following 2 years, the lesion continued to increase in size despite topical antimicrobials and corticosteroids. He was evaluated by a dermatologist and had a lesional biopsy 5 years prior to the presentation which showed perivascular/periadnexal variably dense infiltrates of lymphocytes, plasma cells, multinucleated giant cells, and occasional neutrophils with focal necrosis. Grocott’s methenamine silver and acid-fast bacillus (AFB) stains were negative, T-cell gamma rearrangement testing was negative, and a diagnosis of unilesional granulomatous mycosis fungoides was favored by the dermatopathologist. Repeat skin lesion biopsy was done the following year and again favored unilesional mycosis fungoides. Aerobic, anaerobic, AFB, and fungal cultures were all negative. The patient was treated with oral methotrexate, and the skin lesion resolved over the next year. The patient had additional, unrelated encounters with healthcare 4 years prior to presentation for a motor vehicle accident, with negative computed tomography (CT) head and chest radiograph, and for migraines, with a negative brain MRI. Approximately 1 year prior to presentation, the patient developed left leg pain, edema, and inguinal adenopathy. MRI and positron emission tomography/computed tomography showed ^18^F-fluorodeoxyglucose uptake in the myofascial and periosteal compartments of the left thigh as well as nearby lymph nodes. At that time, he had developed fevers, weight loss, and night sweats. Lymph node biopsy done 7 months prior to presentation was consistent with HHV-8-negative multicentric plasma cell Castleman’s disease. Testing for tuberculosis, *Coccidioides,* and HIV was negative. He had a partial response to siltuximab, prednisone, and then rituximab.

Three weeks prior to presentation, the patient developed new, small, dark lesions on his left anterior thigh and left lateral calf. The patient reported sitting with his feet in a creek around the same time as the development of these lesions on his leg, although the timing is unclear. He had traveled to Mexico a few weeks prior and had a pet dog. Otherwise, he had no other history of travel or other unusual exposures to soil, water, or animals. One week before the presentation, he developed night sweats, followed by chest pain, mild dyspnea, and non-productive cough. Upon presentation, he was afebrile and was not hypoxic or tachypneic and had no neurologic symptoms. By that time, he had been on a corticosteroid wean for 6 months and was on 60 mg of prednisone. He had last received a dose of rituximab 2 weeks prior to presentation.

**Fig 1:**
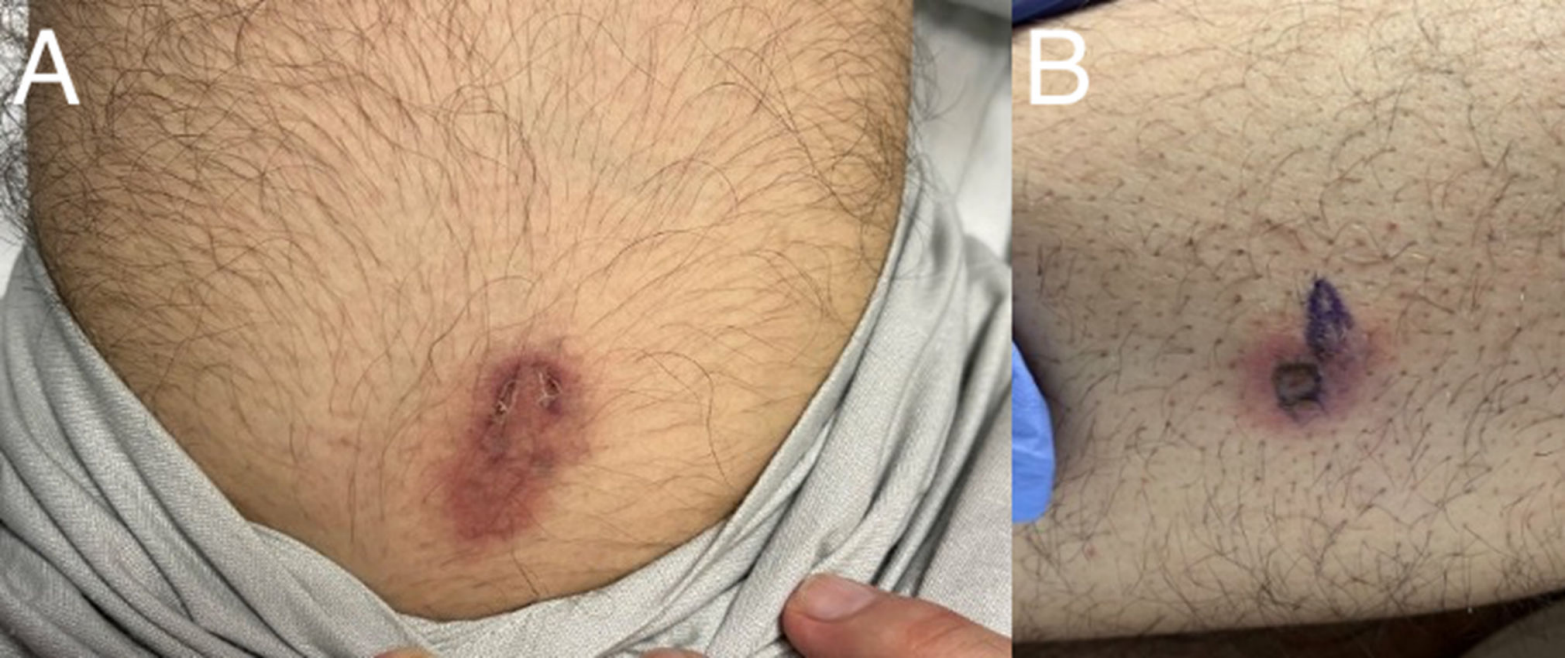
(A) Skin lesion on left thigh. (B) Second skin lesion on distal left lower extremity prior to biopsy. *Balamuthia* forms were observed on subsequent skin biopsy.

**Fig 2:**
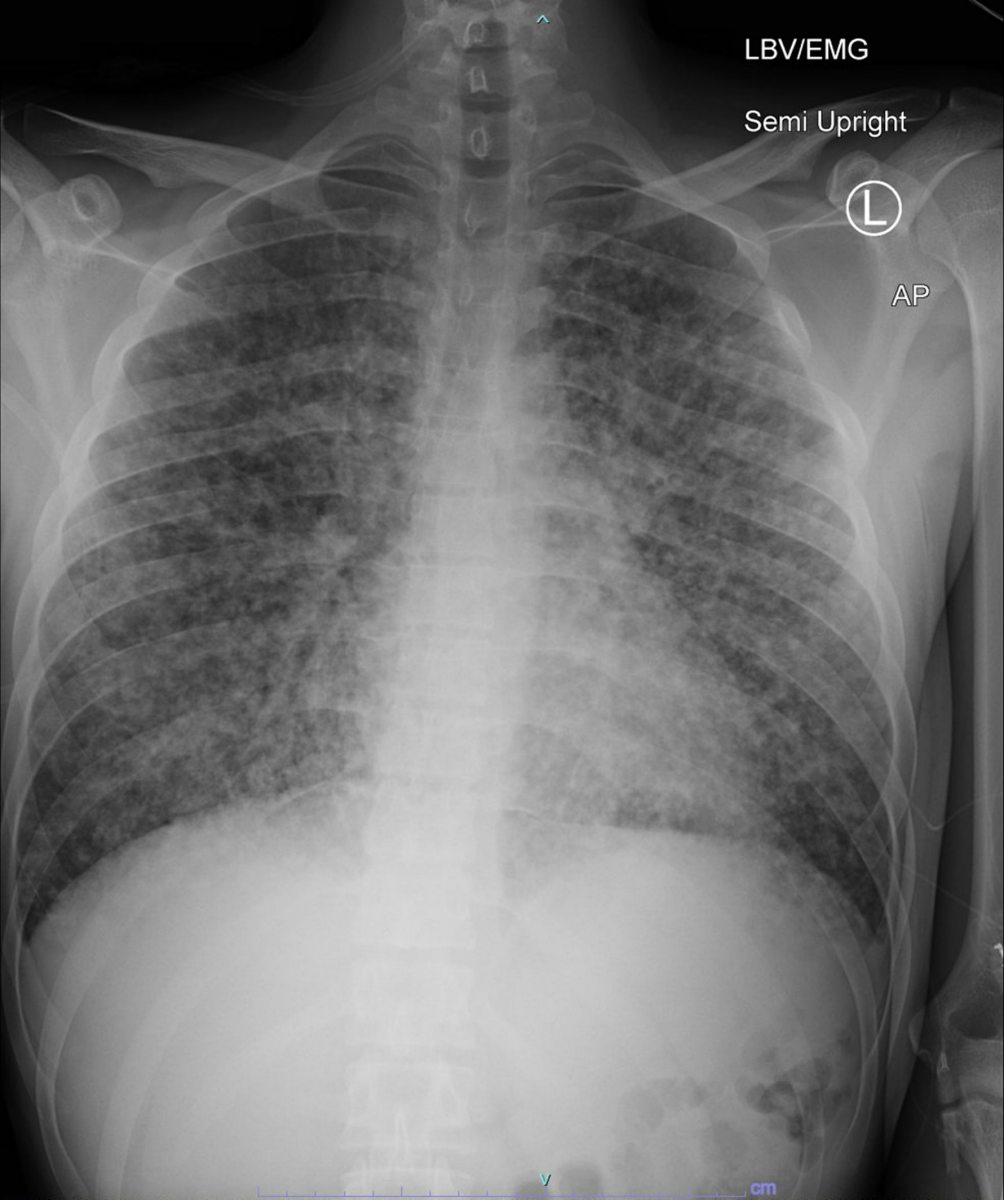
Plain radiograph of the chest demonstrating scattered small pulmonary nodules with a random distribution consistent with miliary pattern of a hematogenous spread.

**Fig 3:**
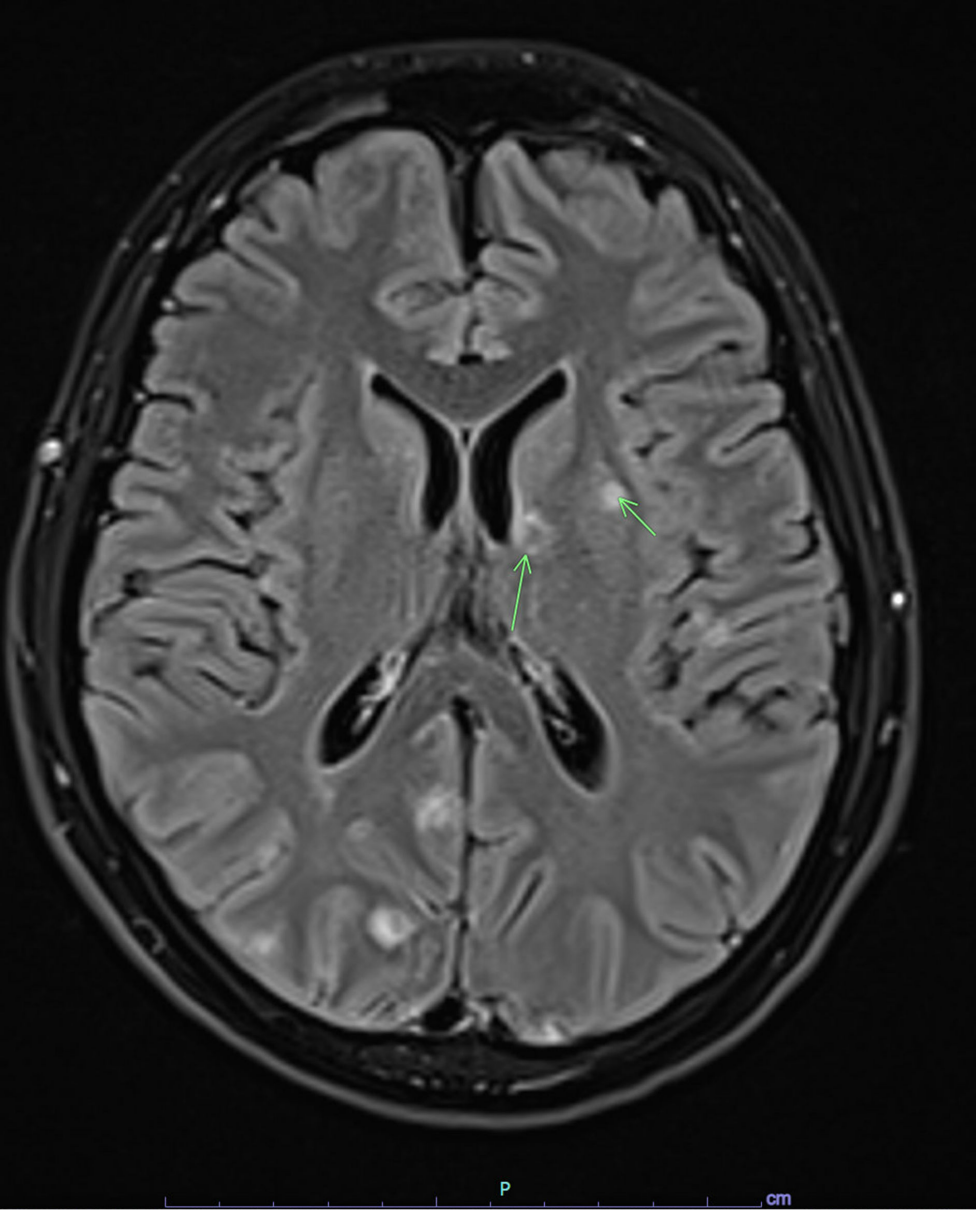
Magnetic resonance imaging of the brain with contrast demonstrating numerous small foci of enhancement at the brain surface within the sulci and throughout the bilateral cerebral hemispheres (arrows point to lesions).

**Fig 4:**
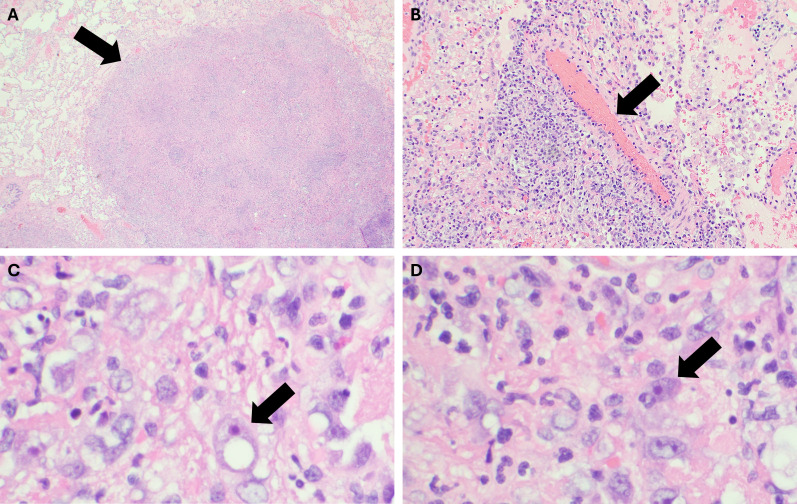
Lung biopsy (hematoxylin and eosin). (A) Acute necrotizing inflammation with abscess formation (arrow pointing to abscess). (B) Neutrophilic vasculitis of the pulmonary vessels (arrow pointing to pulmonary vessel). (C and D) High power view of *Balamuthia mandrillaris* trophozoites (arrows) with prominent nucleoli and heterogenous vacuolated cytoplasm.

Physical exam showed two ovoid, non-blanching, slightly tender violaceous lesions on his left anterior thigh and left lateral calf (Fig. 1). Chest radiograph (Fig. 2) and CT scan showed a miliary pattern of disease. CBC with differential showed 12.9 k/µL white blood cell (WBC) count (normal range: 4.5–13.0 k/µL) and 11.56 k/µL neutrophils (1.80–8.00 k/µL) with normal hemoglobin and platelets. Creatinine, electrolytes, and aminotransferases were normal. C-reactive protein was elevated to 287 mg/L (≤4.9 mg/L). Blood cultures, sputum cultures, QuantiFERON-TB Gold, *Coccidioides* IgM and IgG by enzyme immunoassay, and HIV fourth-generation antibody/antigen screen were all negative. Biopsy of the skin lesion was performed, and on hospital day 4, it was reported as showing trophozoites consistent with amebic disease. Screening MRI of the brain showed numerous small foci of enhancement within the sulci throughout the bilateral cerebral hemispheres (Fig. 3) and cerebellum. There were also multiple intraparenchymal lesions with rim enhancement throughout all lobes and within the cerebellum. Because miliary amebic disease had not previously been described, and due to concern for polymicrobial infection in an immunocompromised patient, lung biopsy was performed. During the procedure, the lungs were noted to have diffuse white pearly nodules throughout all lobes. Lung biopsy showed acute necrotizing inflammation with abscess formation and abundant amebae (Fig. 4). Stains and cultures for bacteria, AFB, and fungi of the biopsied lung tissue were all negative. Lumbar puncture was performed, and the cerebral spinal fluid (CSF) studies showed 3 WBC/µL (normal: 0–5 WBC/µL), <2 RBC/µL (0–10 WBC/µL), glucose 67 mg/dL (40–70 mg/dL), and protein 27.8 mg/dL (15–40 mg/dL). Lung biopsy tissue, skin biopsy tissue, and CSF were sent to the Centers for Disease Control and Prevention (CDC) for Ameba Identification via real-time PCR testing (CDC test code CDC-10286) in the CDC Water, Sanitation and Hygiene Laboratory. The skin and lung specimens tested positive for *Balamuthia mandrillaris*, whereas the CSF PCR was negative. Per the CDC, this assay has 100% specificity and 100% sensitivity compared to ameba-specific indirect immunofluorescence assays and has a detection limit of one ameba per analyzed specimen. Considering the timing of this diagnosis with the recent diagnosis of Castleman’s disease, re-evaluation of the patient’s lymph node biopsy was requested and did not show any histopathological evidence of ameba.

Upon confirmation of the diagnosis of disseminated balamuthiasis, azithromycin (500 mg IV every 24 hours), fluconazole (700 mg IV daily), flucytosine (2,000 mg PO every 6 hours), pentamidine (240 mg IV every 24 hours), and sulfadiazine (1,500 mg PO every 6 hours) were started on hospital day 4. Miltefosine (50 mg PO three times a day) was added the next day when it became available at the treating hospital. On hospital day 6, the patient developed increasing oxygen requirements and altered mental status. He continued to have progressive respiratory failure and obtundation, and developed fever and acute kidney injury. Given his dismal prognosis, his family elected not to escalate support, and he died on hospital day 17. An autopsy was not performed.

## DISCUSSION

A free-living ameba, *Balamuthia mandrillaris,* has a life cycle consisting of a dormant cyst and an active trophozoite stage. The trophozoite is pleomorphic and contains one vesicular nucleus with one or more prominent, dense, and central nucleoli (6, 27). *B. mandrillaris* has a double-walled spherical cyst that measures an average of 15 µm (6). The average size of the trophozoite is around 30 µm but can measure anywhere from 12 to 60 µm. Only trophozoites were observed on histopathology sections of specimens in this case. In biopsy specimens, organisms can be mistaken for histiocytes (3). Immunohistochemical staining, real-time PCR, and next-generation sequencing can aid in making the diagnosis (2, 3, 28).

To our knowledge, this is the first human case of balamuthiasis presenting with miliary disease. *Balamuthia mandrillaris* pulmonary miliary disease has been previously described in a dog and a tiger (29, 30). Among human cases of balamuthiasis, there have been six with confirmed pulmonary involvement described in the literature (Table 1). Five of the six resulted in death, and the outcome of one case is unknown. Two had granulomas with organisms seen on autopsy (10–18). An additional seven cases had pulmonary findings suspicious for *B. mandrillaris* pulmonary disease (Table 1). Findings such as granulomas, radiographic pulmonary nodules, and infiltrates were described; however, an infectious cause of the findings was not confirmed in these reports (2, 8, 19–24). It is important to note that coinfection or other concomitant causes of such findings may be present, as was seen in a patient who died of CNS infection but had mycobacteria observed on acid-fast staining of lung tissue (31).

**TABLE 1:** Confirmed or suspected cases of *Balamuthia mandrillaris* with pulmonary involvement^*a*^

Year	Classification	Geography	Age/sex	Ethnicity	Pulmonary findings	Other sites	Outcome	Time to death after CNS symptoms (skin)	Confirmatory test	Therapy
1991 (17)	Confirmed	Japan	78 y/F	Japanese	Granulomas with trophozoites on autopsy	CNS	Died	2 weeks	IIF	No
2003 (16)	Confirmed	CA, USA	2 y/F	Hispanic	Trophozoites seen on autopsy	CNS	Died	3 weeks	IIF	No
2003 (13, 14)	Confirmed	CA, USA	7 y/M	Hispanic	Lung tissue PCR positive on autopsy	CNS	NR	NR	PCR, IIF	NR
2003 (10–12)	Confirmed	UK/Bolivia	32 y/M	Hispanic	Necrotic lesions with trophozoites on autopsy	CNS/skin	Died	17 days (12 months)	Culture, PCR, IIF	Yes
2009 (15)	Confirmed	CA, USA	64 y/M	Hispanic	Lung tissue PCR positive on autopsy	CNS	Died	12 days	PCR, IIF, serology	NR
2017 (18)	Confirmed	Japan	79 y/M	Japanese	Lung tissue PCR and IHC positive on autopsy	CNS/skin	Died	13 months	IIF, PCR	NR
2024	Confirmed	AZ, USA	17 y/M	Hispanic	Miliary lesions, trophozoites on biopsy	CNS/skin	Died	32 days (38 days)	PCR	Yes
1988 (2, 20)	Suspected	Venezuela	7 y/M	Hispanic	Interstitial pneumonitis	CNS/eye	Died	5 days (2 years)	IIF	No
1994 (24)	Suspected	AZ, USA	27 m/M	NR	Granulomas without trophozoites on autopsy	CNS	Died	16 days	IIF	No
1999 (8)	Suspected	Peru	NR	NR	Granulomas without trophozoites on autopsy	NR	NR	NR	IIF	NR
2006 (19)	Suspected	Chile	5 y/F	Hispanic	Granulomas without trophozoites on autopsy	CNS/skin	Died	5 months (12 months)	Microscopy	No
2012 (23)	Suspected	SC, USA	82 y/M	NR	Multiple nodules on imaging	CNS/skin	Died	9 days (15 months)	PCR, IIF	Yes
2020 (21)	Suspected	China	54 y/M	Chinese	Multiple nodules on imaging	CNS/skin	Died	15 days (unknown)	NGS	Yes
2023 (22)	Suspected	China	64 y/F	Chinese	Consolidation and effusion on imaging	CNS	Died	22 days	NGS	No

^*a*^ If the date of occurrence is unknown, then year indicates the date of publication. NGS, next-generation sequencing; IIF, indirect immunofluorescence assay; NR, not reported.

When *B. mandrillaris* causes CNS infection, mortality approaches 90%. Skin infection may precede CNS infection, sometimes years prior to the development of neurological symptoms, but many CNS infections occur without any cutaneous involvement. Additionally, skin lesions can sometimes resolve without targeted therapy (3, 5). Skin and other lesions can appear similar to those of other disease entities, and a high index of suspicion is needed to make the correct diagnosis, which is often delayed. Many cases of confirmed *B. mandrillaris* infection were initially diagnosed as common bacterial, mycobacterial, and fungal infections, whereas other cases were mistaken for non-infectious causes of skin or CNS lesions such as sarcoidosis, necrobiosis lipoidica, granuloma annulare, rosacea, keloids, cutaneous lymphoproliferative disorder, lymphomatoid granulomatosis, granulomatosis with polyangiitis, acute demyelinating encephalomyelitis, and CNS malignancy. Many received immunosuppressive therapy to treat these diseases. To our knowledge, there have not been reports of *B. mandrillaris* infection misdiagnosed as Castleman’s disease or mycosis fungoides, although mycosis fungoides is often in the differential diagnosis for granulomatous skin lesions (23, 32–38).

It is unclear if our patient’s initial skin lesions from 7 years and 5 years prior to presentation were due to *B. mandrillaris*. No organisms were seen on histopathological examination at the time, but molecular testing was not requested, and the samples were not available for re-evaluation. Re-evaluation of the patient’s lymph node biopsy (7 months prior to presentation) was performed and did not show any histopathologic evidence of amebae.

Rapid diagnosis and targeted therapy may lead to improved outcomes. In a patient with miliary lung disease, *B. mandrillaris* should be included in the differential diagnosis, and appropriate diagnostic testing should be considered.
